# Desmosomes In Vivo

**DOI:** 10.1155/2010/212439

**Published:** 2010-06-24

**Authors:** David Garrod

**Affiliations:** Faculty of Life Sciences, University of Manchester, Michael Smith Building, Oxford Road, Manchester M13 9PT, UK

## Abstract

The structure, function, and regulation of desmosomal adhesion in vivo are discussed. Most desmosomes in tissues exhibit calcium-independent adhesion, which is strongly adhesive or “hyperadhesive”. This is fundamental to tissue strength. Almost all studies in culture are done on weakly adhesive, calcium-dependent desmosomes, although hyperadhesion can be readily obtained in confluent cell culture. Calcium dependence is a default condition in vivo, found in wounds and embryonic development. Hyperadhesion appears to be associated with an ordered arrangement of the extracellular domains of the desmosomal cadherins, which gives rise to the intercellular midline identified in ultrastructural studies. This in turn probably depends on molecular order in the desmosomal plaque. Protein kinase C downregulates hyperadhesion and there is preliminary evidence that it may also be regulated by tyrosine kinases. Downregulation of desmosomes in vivo may occur by internalisation of whole desmosomes rather than disassembly. Hyperadhesion has implications for diseases such as pemphigus.

## 1. Introduction

Desmosomes are intercellular junctions that are uniquely able to provide very strong intercellular adhesion [1, 2]. This is especially important in tissues that are subject to mechanical stress such as epidermis and cardiac muscle. They are present in the lowliest, jawless vertebrates, hagfishes, but appear to be absent from our nearest chordate ancestors [3–7]. It is probable, therefore, that they have played a key role in vertebrate evolution by contributing to a strong integument and a powerful heart. 

**Figure 1:**
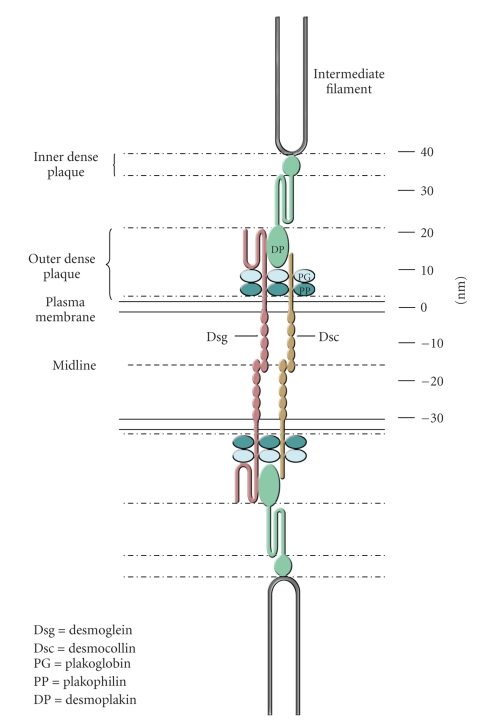
Schematic model of a desmosome showing the relative positions of the major desmosomal components. (See Figure 4 for an electron micrograph of desmosomes.) The scale on the right-hand side indicates distance in nanometres. The figure is largely based on the immunogold labelling experiments of North et al. [8]. Reproduced from the study by Garrod and Chidgey in [1].

Desmosomes are composed of a small number of well-defined molecular components (Figure 1). These are their adhesion molecules, the desmosomal cadherins desmoglein and desmocollin, the plakin desmoplakin that links the adhesion molecules to the intermediate filaments (IFs), and the armadillo proteins plakoglobin and plakophilin that link the adhesion molecules to desmoplakin and appear to regulate desmosomal assembly and size. For details please see recent reviews in [1, 9–16].

## 2. Epithelial Cells In Vivo: Some Myths and Specific Junctional Considerations

The major topic of this review will be a consideration of how desmosomes function in vivo with particular reference to their adhesive properties. It is relatively easy to study cell behaviour and function in culture. Cellular organelles are readily visualized, extracted, and subjected to a wide range of analytical techniques. The results obtained are often striking, but are they relevant in vivo? At worst they may be complete artefacts; at best they may suggest a relevant in vivo mechanism, but this needs to be demonstrated. An ideal but perhaps unrealistic goal would be for all discoveries made in tissue culture to be followed by attempts to show their relevance in vivo. 

We have a striking example of this in the field of cell adhesion. Focal contacts are small, usually elongated structures of the order of 1 *μ*m in length where the basal surfaces of cells spread on solid surfaces in culture approach the substratum to a separation of about 15 nm [17]. Cytoplasmically they provide the insertion points for actin stress fibers. They are easily visualized by interference refection microscopy, electron microscopy, or fluorescence imaging of any one of a prodigious number of proteins that are associated with them [17, 18]. They are fascinating structures but what is their relevance in vivo? This issue seems hardly to have been addressed, though it is beginning to be [19, 20]. An extreme view, hopefully incorrect, would be that they are culture artifacts and that their study does little to elucidate relevant in vivo mechanisms of cell adhesion. 

Desmosomes occur predominantly in epithelial cells. In vivo these cells contribute to epithelia, which are continuous or confluent cell sheets providing dynamic barriers between body compartments. The confluence of the sheet is vital; without it the barrier function of the epithelium cannot be maintained. If confluence is broken in a healthy individual, the epithelium repairs itself to restore the barrier as rapidly as possible, as most easily and dramatically seen in epidermal wound healing. 

**Figure 2:**
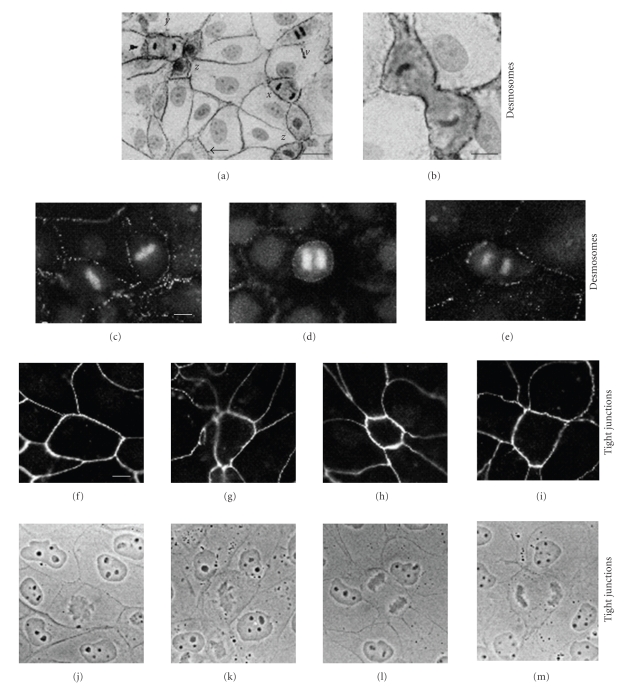
Epithelial cells retain junctional contact during cell division. (a) and (b) MDCK cells stained with monoclonal antibody to desmoplakin by the ABC technique and counterstained with haematoxylin. (a) Cells at various stages of division are shown: *w*, anaphase; *x*, telophase; *y*, early cytokinesis; *z*, advanced cytokinesis. All dividing cells show prominent peripheral staining for desmoplakin. In some cases (e.g., small arrowhead), the desmoplakin staining is punctate and not obviously confined to the cell periphery. However, similar staining is commonly seen in nondividing cells (e.g., large arrowhead) and appears to be due to oblique viewing of the cell interfaces. (b) High-power photographs of similar cell in advanced cytokinesis. Note the prominent peripheral desmoplakin staining, even at the borders of the cleavage furrow. Although the cytoplasm is generally more darkly staining than that of neighbouring non-dividing cells, this does not indicate desmosome internalisation (see following fluorescence micrographs). Bars: 20 m (a) and 10 m (b) (c)–(e) MDCK cells stained with monoclonal antibody to desmoplakin and propidium iodide to show chromosomes. (c) Two cells in metaphase, (d) cell in early anaphase, and (e) cell in late anaphase. All dividing cells show peripheral punctate staining for desmoplakin, comparable to that seen in non-dividing neighbours. However, no punctate staining indicative of desmosome internalisation is present in the cytoplasm of the dividing cells. Bar: 10 m. (f)–(i) MDCK cells stained with monoclonal antibody to tight junction protein ZO-1. (j)–(m) Corresponding phase-contrast images. The cell in (F,J) is in prophase, that in (g) and (k) in metaphase and those in the remaining pictures in telophase. Each dividing cell is surrounded by a complete ring of ZO-1 staining and shows no evidence of junction internalization or disruption. Dividing cells in epidermis and intestine also showed retention of junctions by electron microscopy. Bar: 10 m. Reproduced from the study by Baker and Garrod in [21].

Maintenance of confluence and barrier function appears to be a compulsive behaviour of epithelial cells. Intercellular junctions, including desmosomes, are key contributors to this process. Two commonly held myths in cell biology are that (i) cells lose adhesion during cell division and (ii) cell division is inhibited by intercellular contact, the so-called “contact inhibition of cell division”. The former notion seems to have arisen from watching isolated fibroblasts round up when they divide in culture. Certainly cell division necessitates a change of shape and some remodelling of cell contacts. But epithelial cells in confluent sheets, both in culture and in vivo, retain tight and adherens junctions and desmosomes throughout cell division [21] (Figure 2). If this were not the case, the barrier function of the epithelium would be compromised every time a cell divided. The drive to maintain confluence and barrier function is also manifest when cells in epithelial cell sheets undergo apoptosis. The dying cell is often extruded from the sheet and this would leave a hole if nothing were done about it. In fact neighbours of the extruded cell move to fill the gap, zipping up junctions as they go and presumably enabling a seamless continuity of barrier function [22]. Although junctions are maintained throughout these processes, it is likely that modulation of junctions in localised regions of the cell surface may be required in order to facilitate cell shape changes. 

Maintenance of cell-cell contact during cell division graphically demonstrates that division of epithelial cells is not inhibited by intercellular contact. A careful analysis of fibroblast division has shown that this is also not inhibited by contact [23]. A moment's thought about cells in vivo suffices to conclude that the very existence of multicellular organisms would be impossible if there were such a thing as “contact inhibition of cell division”. In vivo all epithelial cells are in continuous or confluent cell sheets, yet many epithelia exhibit rapid cell turnover involving continuous cell division to replenish lost cells. How would this be possible if cell division were inhibited by intercellular contact? In saying this it is also recognised that cell-cell adhesion can contribute to the regulation of the plane and, in some cases, the rate of cell division and the latter may be particularly important in some situations, for example, in cancer [24–27]. 

The key reason for retention of junctions during cell division is that epithelial barriers must be maintained at all costs. Small skin wounds can be tolerated temporarily provided that the hole is rapidly plugged by a clot and that the epidermal covering is restored with reasonable alacrity. When the epidermal barrier is severely compromised, as in extensive burning, the consequences are lethal. Intercellular junctions are vital for the barrier as illustrated by some human mutations and engineered defects in mice [28–32] in which loss of junctional function compromises the epidermal barrier causing rapid neonatal death. Less obvious but equally important is the maintenance of internal epithelial barriers. Diminished barrier function in the gut gives rise to inflammatory bowel disease and airway epithelial barrier is compromised both in asthma and by invading allergens, leading to enhanced allergen delivery and respiratory allergy [33, 34]. 

When interpreting studies on cultured epithelial cells careful attention should be given to the degree of culture confluence under which the observations were made. Many such observations are made on subconfluent cells. This is fine provided it is recognised that subconfluent epithelial cells are in an abnormal or “activated” state broadly equivalent to cells at the edge of a wound. The behaviour of such cells is fundamentally different from the behaviour of epithelial cells in cell sheets in culture or, most important, in vivo. A simple example will illustrate this. Clumps of subconfluent cultured epithelial cells such as MDCK treated with hepatocytes growth factor (HGF) (scatter factor) will scatter, undergoing epithelial-mesenchymal transition (EMT) and downregulating their intercellular junctions [35, 36]. Other growth factors produce similar effects with other types of epithelial cell. However, treatment of confluent epithelial cells with HGF or a range of other growth factors has no effect whatsoever on cell-cell adhesion [37]. Thus, although scattering by growth factors and EMT are fascinating behaviours, some change in the epithelium equivalent to wounding or “activation” would appear necessary for them to become relevant to an epithelium in vivo. However, the essential role for such signalling mechanisms in specific in vivo aspects of cell migration, for example, limb bud development, is clear [38]. 

For many purposes confluent cells are less easy to study than subconfluent cells. However, confluence is essential if it is desired to approach in culture the “normal” situation for epithelial cells in vivo.

## 3. Calcium-Induced Desmosome Assembly and Its Significance

It is frequently stated that desmosomal adhesion is calcium dependent. This is because of the following. (a) The desmosomal adhesion molecules, desmocollin and desmoglein, are members of the cadherin family and bind calcium; (b) desmosomes (and other junctions) do not form when cells are cultured at an extracellular calcium concentration of <0.1 mM; (c) when the calcium concentration is raised, desmosomes form rapidly between cells cultured as in (b); (d) desmosomal adhesion is lost by subconfluent or early confluent cells when extracellular calcium is depleted. 

This is all indisputable but it must be realised that desmosomes in tissues do not behave and are not regulated in this way. In the first place the calcium concentration of tissue fluid is rigorously maintained at about 1 mM. Variations from this level have serious consequences for many bodily functions including nerve conduction and muscle contraction. It is therefore unlikely in the extreme that variations in extracellular calcium concentration have anything to do with the regulation of cell adhesion in vivo. At worst, observations on junctional changes induced by “calcium switching” in culture may be completely artefactual. On the other hand “calcium switching” may be a suitable model for junction assembly in vivo, but this needs to be rigorously tested. 

Calcium-induced desmosome assembly appears to be a simple process of stabilisation of adhesion. Cells cultured in low-calcium medium (LCM) synthesise desmosomal components [39, 40]. The desmosomal cadherins, or assemblies of desmosomal proteins in the form of half-desmosomes, are transported to the cell surface where they are unable to participate in adhesive binding [39, 41]. They are therefore internalised and degraded, so in LCM they have short half-lives. Desmoplakin also has a short half life in LCM but usually appears to remain in the cytoplasm in punctate form associated with the cytoskeleton, or in a more diffuse pool [42, 43]. When the calcium concentration is raised, adhesion is triggered and desmosomes assemble at cell interfaces, a process that continues for up to 36 hours in MDCK cells [42–46]. Such desmosomes become relatively stable structures; nevertheless desmocollin remains fairly mobile [47]. 

Adhesion is triggered because a calcium concentration of ca. 1 mM is necessary to maintain cadherin extracellular domains in an extended configuration such that they are adhesion competent [48]. In vivo every cadherin molecule emerging onto the cell surface is instantly exposed to such a concentration of calcium (intracellular calcium is in the M range) and therefore will immediately become adhesion competent. Thus the only calcium switch in vivo probably occurs when a molecule emerges from the intracellular into the extracellular environment. 

A similar argument indicates that calcium depletion does not induce loss of junctional adhesion in vivo. On the other hand, depletion or chelation of extracellular calcium can be used as an experimental assay of desmosomal adhesiveness. Calcium depletion applied to subconfluent or early confluent epithelial cells in culture induces a genuine loss of desmosomal adhesion, which is a splitting of desmosomes through the intercellular region, resulting in the formation of half desmosomes that are subsequently internalized by the cells [49, 50]. This presumably occurs because of the reverse of the process described above; the cadherin extracellular domains lose their extended, adhesion-competent configuration. However, this type of “calcium-dependent” desmosomal adhesion, though commonly encountered with cells in culture, appears to be rare in vivo, only being encountered in special situations where cells are “activated” for tissue modelling or remodelling, such as embryonic development or wound closure.

## 4. Desmosomes Form an Insoluble Complex with Intermediate Filaments

Fey et al. sequentially extracted confluent epithelial cells to generate the nuclear matrix-intermediate filament (NM-IF) scaffold [51]. This is the cytoskeletal network remaining after extraction with the nonionic detergent Triton X-100, high salt, RNase, and DNase. The NM-IF contains junctional components including desmosomes, as verified by electron microscopy. To dissolve the NM-IF it was necessary to treat it with sodium dodecyl sulphate (SDS) and a reducing agent or urea. Even when isolated from intermediate filaments, desmosomes remain highly insoluble. Thus desmosomes isolated from bovine nasal epithelium by treatment with citric acid-sodium citrate buffer at pH2.6, which dissolves keratin filaments, have to be dialysed for hours against SDS-containing buffer in order to dissolve them [52, 53]. 

Such insolubility is an indicator of the toughness of desmosome that fits them for their role of extremely strong adhesion. From the biologist's viewpoint it makes it extremely difficult to study protein-protein interactions in desmosomes because dissolving them disrupts such interactions. Many biochemical procedures such as pull-down assays carried out on various soluble fractions of cultured cells must therefore be interpreted with extreme caution when applied to desmosomal components because they do not provide information relating to intact desmosomes. The interactions that they reveal apply to upstream events, that is, prior to desmosome assembly, or downstream events, that is, after proteins have left the desmosome. They may also reflect interactions within desmosomes but it should not be assumed that they do without further proof.

## 5. Desmosome “Modulation” Requires Epithelial Confluence

**Figure 3:**
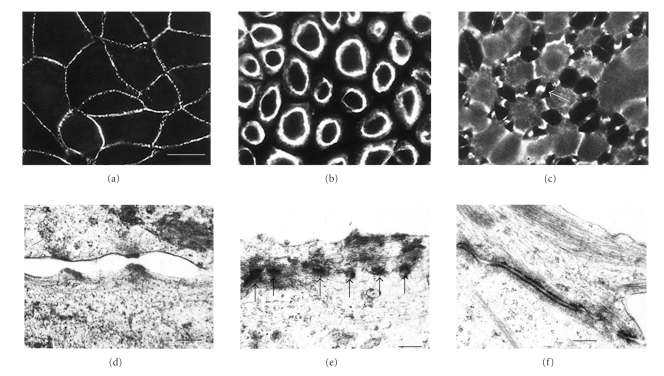
Desmosomes of MDCK cells can be calcium-dependent or calcium-independent. (a) Desmosomes of MDCK cells in confluent culture stained with monoclonal antibody to desmoplakin. Note that the desmosomes are located at the cell peripheries and that the staining is generally punctate. (b) A monolayer that has been cultured at confluent density for 24 hours in SM and then treated with LCM-EGTA for 90 minutes, showing loss of intercellular contact and of desmosomal staining from the cell peripheries. This is indicative of calcium-dependent desmosomes. (c) A 6-day-confluent monolayer treated with LCM-EGTA for 90 minutes, showing partial loss of intercellular contact but persistence of joining processes with intense desmosomal staining (e.g., arrow). This is indicative of calcium-desmosomes. Bar, 20 m. (d) When subconfluent or 1-day-confluent cells are treated with LCM-EGTA adhesion is lost and desmosomal halves separate. The half desmosomes are then internalised ((e), arrows). (f) By contrast when 6-day-confluent cells are treated with LCM-EGTA desmosomes remain adherent and accumulate at the cell surface between interconnecting cell processes. Bars: 0.3 m in (d) and (f); 0.4 m in (e) (a)–(c). Reproduced from the study by Wallis et al. in [37]. The isoform of protein kinase (c) is involved in signalling the response of desmosomes to wounding in cultured epithelial cells. (d)–(f) Reproduced from the study by Mattey and Garrod in [50].

Using the calcium chelation assay it has been shown that, while the maintenance of desmosomal adhesion is initially calcium dependent, it matures to become functionally calcium independent [37, 45, 46, 54] (Figure 3). Mature, calcium-independent desmosomes do not lose adhesion and split in the intercellular space when extracellular calcium is depleted. Although other types of junction, the tight and adherens junctions, may break down leading to substantial loss of intercellular adhesion, the desmosomes remain adherent and continue to bind cells together [37, 50, 54]. This can cause the cells to adopt a stellate appearance since, although largely rounded, they remain attached at points where desmosomes accumulate during the retraction process [37] (Figure 3). 

Development of calcium independence requires culture confluence. If cells are maintained at subconfluent density, calcium independence does not develop [37]. In confluent epithelial cell cultures the onset of calcium independence is gradual, in the sense that it takes several days for all of the cells to acquire mature desmosomes [37, 54]. Slow junctional maturation is also found in tight junctions, which generally take several days of confluent culture to develop so that transepithelial electrical resistance is maximal [57–59]. 

In order to maintain desmosomal calcium-independence, culture confluence must also be maintained. If a cell sheet in which calcium-independence has reached 100% is scratch wounded, the cells at the wound edge reacquire calcium dependence within about an hour [37]. This effect is then propagated away from the wound edge through the cell sheet. No evidence could be found for the involvement of extracellular diffusible factors or for the release of cytoskeletal tension in this propagation. The most likely explanation is that some form of cell-cell communication via gap junctions, such as intercellular calcium signals, is involved, but this has yet to be demonstrated.

## 6. Calcium-Independence Is the Normal State for Desmosomes In Vivo

**Figure 4:**
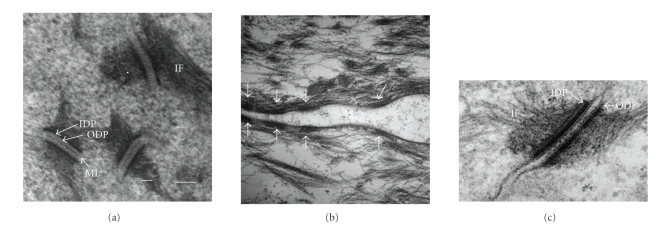
Transmission electron micrographs of desmosomes in mouse epidermis. Normal (a) and wound-edge (b) mouse epidermis after exposure to LCM-EGTA for 6 hours (a) and 1 hour (b). Note that the desmosomes in (a) are intact and two that are sectioned precisely transversely exhibit entirely unaltered structure, whereas in (b) the desmosomal halves (arrows) have lost adhesion and separated (compare with Figure 3(d)). Thus the desmosomes in (a) are calcium independent and those in (b) are calcium dependent. (c) An example of transversely sectioned desmosomes from untreated wound-edge epidermis showing absence of midline but plaque structure appears unchanged at this resolution. ML, midline; ODP, outer dense plaque; IDP, inner dense plaque; IF, intermediate filaments. Bar: 0.1 m. Reproduced from the study by Garrod et al. in [55].

Epithelia of animal tissues are confluent cell sheets and remain so unless wounded or diseased. If the above observations on the adhesive properties of desmosomes in cell culture are of in vivo relevance, it would be expected that desmosomes in vivo should generally be calcium independent. That this is the case has been rigorously demonstrated for the epidermis by exposing small pieces of excised mouse epidermis to EGTA for several hours and examining junctional structure by electron microscopy [55]. It was found that all 200 desmosomes examined retained adhesion and apparently unaltered structure after 6 hours of incubation in EGTA (Figure 4). Previously it had been found in a wider survey of adult mouse tissues by immunofluorescence that desmosome calcium-independence was the norm [37]. In addition to epiderm are these included trachea, oesophagus, tongue, liver, and cardiac muscle. Here it was shown that adherens junctions (E-cadherin staining) were internalised upon EGTA treatment, so there is no doubt that the chelating agent was able to penetrate the tissues. 

It was surprising to find that calcium-independence was so all-pervading; no calcium-dependent desmosomes were found, even in the basal layer of epidermis. Presumably desmosome assembly is likely to be a continuous process in epidermis because of cell turnover. This may indicate that desmosomal adhesion matures very rapidly in vivo or that newly formed desmosomes are extremely rare and therefore difficult to find. In order to determine whether desmosomal calcium-dependence occurs at any stage in vivo we have examined desmosomes in developing mouse epidermis (T. E. Kimura, A. J. Merritt, and D. R. Garrod; unpublished). Using both immunofluorescence and electron microscopy to assay the results, we found that epidermal desmosomes remained entirely calcium dependent until E12 (embryonic day 12) but by E14 > 75% of epidermal cells possessed calcium-independent desmosomes. By E19 this had increased to almost 100%. In contrast, adherens junctions remained calcium dependent throughout. Thus it appears that desmosomal adhesiveness is developmentally upregulated. 

Our results should be compared with earlier results on tissues of the frog Rana pipiens that were exposed to the nonspecific chelating agent EDTA and examined by electron microscopy [60]. Borysenko and Revel showed that desmosomes of stratified squamous and glandular epithelia were insensitive to EDTA but that those of simple columnar epithelia were EDTA-sensitive. The latter result was in agreement with that of a study on oxyntic cells of the gastric glands, in which it was found that EDTA induced desmosomal changes were reversed by readdition of calcium [61]. Since our studies have shown that mammalian simple epithelial cells such as MDCK readily develop calcium-independent desmosomes, it appears that mammals and frogs may differ in this respect. It is interesting that the oxyntic cell desmosomes studied by Sedar and Forte showed no intercellular midlines, whereas those of the frog epidermis, shown by Borysenko and Revel, had very distinct midlines (see below for the significance of this observation).

## 7. Desmosomes Revert to Calcium-Dependence in Wounded Epidermis

Culture experiments suggest that wounding a confluent cell sheet causes desmosomes to revert from calcium independence to calcium dependence. In order to determine whether similar changes occur in vivo, wound-edge epithelium from mouse epidermis was incubated in EGTA and the desmosomes examined by electron microscopy [55]. It was found that 53.3% of such desmosomes were calcium dependent by 48 hours after wounding and 63.8% by 72 hours (Figure 4). Thus the change in desmosomal adhesiveness shown on wounding by simple epithelial cells in tissue culture is also an in vivo phenomenon in the epidermis indicating that cell culture potentially provides a good model for desmosome regulation in tissues.

## 8. Calcium-Independent Desmosomes Are Hyperadhesive

Because calcium independence makes desmosomes difficult or impossible to dissociate, it seemed likely that it represents a strongly adhesive state. In order to test this we compared the adhesiveness of dispase-detached sheets of epidermal keratinocytes (HaCaT) with calcium-dependent and calcium-independent desmosomes using a fragmentation assay [54]. So as to be sure that we, really were comparing adhesiveness we confirmed by electron microscopy that the fragmentation was occurring by splitting desmosomes in the intercellular region rather than by breaking cells. Differential cell breaking could also increase the number of sheet fragments in response to mechanical stress, but would indicate differences in other cellular properties, such as cytoskeletal changes, rather than adhesiveness (see below for disease situations where cell breaking occurs). The results showed that sheets with calcium-independent desmosomes were considerably more cohesive than those with calcium-dependent desmosomes. Moreover, this greater cohesiveness persisted in the presence of EGTA, indicating that it was a truly calcium-independent phenomenon. 

The greater adhesiveness of calcium-independent desmosomes has been termed “hyperadhesion” because it is a unique and very important property. It is unique because it does not appear to be a feature of the other major adhesive junction, the adherens junction, which is always calcium dependent according to all reports and to our experience with cultured cells and tissues. It is very important because it is fundamental to the strength and integrity of vertebrate tissues. In contrast to hairy animals human beings are surrounded by an extremely thin epithelial layer, the epidermis, and this is their only protection against severe environmental stresses such as physical abrasion and dehydration. It would not be able to do this without desmosomal hyperadhesion, which binds its cells tightly together into a tough, cohesive epithelium. In addition they have cardiac muscle that can generate a pressure of almost 1/3 atmospheric, again demanding tight cohesion between cardiac myocytes. 

It has long been axiomatic that desmosomes provide strong adhesion but it has never before actually been demonstrated that this strong adhesion is a regulable property dependent on desmosome maturation and requiring calcium-independent adhesive binding by cadherins. The assumption of strong adhesiveness has been based on a number of considerations. Firstly, desmosomes are most abundant in tissues such as epidermis where resistance to physical stress is essential. Secondly, desmosomal adhesion is the intercellular link in the desmosome-intermediate filament complex, which forms a cytoskeletal network extending throughout tissues that contain it. Such a bracing network needs equivalent strength at every point or it would not function [1]. Thirdly, several human diseases that affect desmosomal components weaken the tissues that are most affected, such as the epidermis and the heart [16, 62, 63]. Fourthly, several constitutive or conditional deletions of desmosomal genes from mice also result in weakening of the epidermis and/or the heart [32, 64–67]. The demonstration of hyperadhesion provides experimental confirmation of a long-held belief.

## 9. Acquisition of Hyperadhesion Involves No Change in the Composition of Desmosomes

A simple explanation of hyperadhesion and its regulation might be that one or other of these components is recruited to or lost from desmosomes to regulate this adhesive change. We had previously found that inhibition of protein synthesis with cycloheximide did not prevent the conversion of desmosomes making this explanation unlikely. Because the extent of hyperadhesion in HaCaT cell sheets was easily quantifiable and the desmosomes in these cells could be readily and rapidly converted between the two states (see below), we made qualitative and quantitative comparison between the desmosomal components in cells expressing one state and the other. The cells express plakoglobin, desmoplakin, two isoforms each of desmoglein and desmocollin, and three isoforms of plakophilin. There was no qualitative or quantitative difference in any of the components between cells in the two adhesive states, nor in the localisation of any component to desmosomes at the cell periphery [54].

## 10. Desmosome Structure Suggests a Basis for Hyperadhesion

A striking and unique feature of the structure of desmosomes is the presence of an electron-dense midline between the plasma membranes in the intercellular space [60, 68, 69]. The midline lies at the centre of the adhesive interface between adjacent cells and thus may represent a structural specialisation for hyperadhesion. The other major adhesive junction, the adherens junction, which does not appear to adopt hyperadhesion, notably lacks a midline. A significant finding is that desmosomes in epidermal wound-edge epithelium, which are generally calcium dependent, lack a midline and their intercellular space is ca.10% narrower than that of hyperadhesive, midline-possessing desmosomes of unwounded epidermis [55]. The intercellular space of the calcium-dependent adherens junction is generally reported as being narrower than that of the desmosome. The calcium-dependent desmosomes of embryonic epidermis also lack midlines, but midlines are acquired together with hyperadhesion (T. E. Kimura et al., unpublished). Thus hyperadhesion seems to go together with a wider intercellular space and the presence of a midline, while calcium-dependence seems to be accompanied by a narrower intercellular space with no midline. 

A short paper by Rayns et al. provided the only significant insight into the structure of the desmosomal interspace until very recently [70]. The most noteworthy feature of this structure was its regularity. Infiltration of guinea-pig heart muscle with the electron-dense tracer, lanthanum, revealed a highly regular structure in which the midline appeared as a zigzag that was connected to the plasma membranes by alternating cross-bridges (alternating light and dark parallel lines) of 70–75 periodicity. Tangential sections through these structures revealed arrays of dense particles of 75 periodicity or quadratic arrays of dense particles of 55 centre-to-centre spacing [70]. 

In order to form crystals, molecules must pack into regular arrays. When the crystal structure of the partial EC domain of the classical cadherin, N-cadherin, was published, it was described as an “adhesion zipper” that resembled the ultrastructure of the desmosome rather than the adherens junction [71, 72]. The later publication of the crystal structure of the full-length EC domain of Xenopus C-cadherin produced a model that seemed even more to resemble a desmosome [56]. We were struck by the remarkable similarity between the periodicity found in the C-cadherin structure and that previously reported by Rayns et al. [70]. Homology models for Dsc2 and Dsg2 were generated using the C-cadherin ectodomain as a template. Modelling of the desmosomal cadherins (Dsc2 and Dsg2) in the crystal packing observed for C-cadherin shows that it is feasible to produce a similar array, despite the modest sequence identity between them (ca.30%) [55]. However, some differences in detail between the intermolecular and intramolecular interactions in the desmosomal cadherins may be expected. The final quality of the models for Dsc2 and Dsg2 was compared with the C-cadherin structure. The root mean square deviation for all equivalent carbons was 1.03 for Dsc2 and 1.04 for Dsg2. 

**Figure 5:**
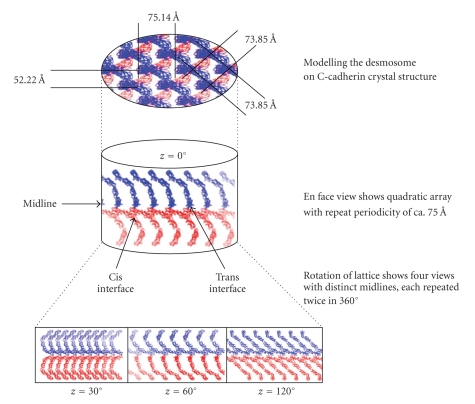
Schematic representation of the Dsc2 ectodomain model 3D array, generated from the crystallographic structure of C-cadherin [56]. In the centre we represented the desmosomal interspace as a cylinder, showing the midline formed by trans and cis interactions between molecules on opposed cell surfaces. Monomers from one cell surface are coloured in red and monomers from the opposed cell surface are coloured in blue. The midline is aligned with the *x*-axis of the cylinder (coincident with the *x*-axis in the crystallographic lattice). Rotation of the 3D array around the *x*-axis by 90° (top) shows a regular lattice with distances between rows of molecules of 73.85 and distances of 75.14 between layers. Rotations around the *z*-axis produce four different views, all of them with a midline dense zone (bottom). These are strand dimer1 at *z* = 0°, “boat” at *z* = 30°, strand dimer 2 (inverse form strand dimer 1) at *z* = 60°, and “zipper” at *z* = 120°. The cis and trans interfaces between distal domains are indicated by arrows. Reproduced from the study by Garrod et al. in [55].

In an attempt to model the Ca^2+^-independent desmosome structure we computed a 3D array of Dsc2 molecules generated according to the crystallographic cell symmetry of C-cadherin [55]. A similar array was generated for Dsg2. In these arrays, the adhesion interfaces are aligned to the *x*-axis (along the crystallographic *x*-axis) (Figure 5 shows the Dsc2 array). The structure seemed to account very well for the desmosomal midline and showed remarkable agreement with the repeating periodicity shown by Rayns et al. [70] Therefore, we proposed that this 3D array is a good model for the highly ordered, quasicrystalline desmosome structure observed in ultrastructural studies. 

A most exciting development has been the study of the extracellular domains of tissue desmosomes by cryoelectron tomography of vitreous sections [73]. This technique enables the visualization of three-dimensional molecular structure under close-to-native conditions. Three-dimensional reconstruction showed a regular array of densities at 70 intervals along the midline, with a curved shape resembling the X-ray structure of C-cadherin. 

How is it possible for desmosomes to alternate between calcium-independent hyperadhesion and calcium dependence without changing their molecular composition? We suggest that the ordered arrangement of the EC domains of the desmosomal cadherins is dynamically variable. In support of this are our observations that the midline structure is not evident in calcium-dependent embryonic and wound-edge epithelial desmosomes. It is probable that such desmosomes have a less ordered arrangement of the EC domains, which therefore appear more diffuse in the electron microscope. Thus we suggest that a type of locking mechanism operates; in the ordered arrangement, the EC domains are locked together giving strong adhesion that cannot be readily dissociated, while in the less ordered arrangement, adhesion is weaker and dissociation is possible. We also suggested that the locked form may involve entrapment of calcium ions, a possible explanation of “calcium independence”. 

An alternative view of desmosome structure is that the desmosomal cadherins in the desmosomal interspace form a series of tangled knots rather than a regular structure [74]. It seems to the author that such an arrangement is less likely to account for the dynamic properties of desmosomes and for their great adhesive strength. The desmosomes studied in this paper were from neonatal mouse epidermis. It is possible that their cadherins appeared irregular because the desmosomes were calcium dependent, though this seems unlikely for two reasons. Firstly they had midlines, which in our experience are invariably associated with calcium independence. Secondly, a study of the developmental regulation of desmosomal adhesion in the mouse epidermis has shown a major change to calcium independence by E14 and by E19: 100% of keratinocytes have calcium independent adhesion, as in the adult (Kimura at al., unpublished) [55]. A more probable explanation is that the desmosomes studied were calcium independent and that the structure was distorted during preparation, which involved freeze substitution and resin embedding.

## 11. An Ordered Intercellular Zone Requires an Ordered Plaque

The cytoplasmic domains of the desmosomal cadherins lie in the desmosomal plaque. An ordered arrangement of their extracellular domains would seem to require an equally ordered arrangement of their cytoplasmic domains and therefore of the plaque in general. Several ultrastructural studies have provided preliminary evidence that the plaque has a lamellar structure parallel to the membrane and a periodic structure at right angle to it. 

Thus, the desmosomal plaque shows filamentous or periodic organisation perpendicular to the plasma membrane and lamellar organisation parallel to it. The former was revealed by early freeze-fracture studies [75–78]. Cryosections of tissue prepared by the Tokyasu method revealed an electron opaque lamina ca. 17 nm from the plasma membrane while negative staining of polyvinyl alcohol-embedded material revealed the presence of at least two laminae in the outer dense plaque [8, 79]. Miller et al. [79] noted transverse periodicity of approximately 2.6 nm in a region probably corresponding to the inner dense plaque (IDP) and North et al. [8] observed fine filaments perpendicular to the membrane in the outer dense plaque (ODP). A remarkably similar picture has emerged from cryoelectron microscopy of rapidly frozen, fully hydrated human epidermis [80, 81]. The desmosomal plaque showed a 10-11 nm region of medium electron density adjacent to the plasma membrane (PM) followed by a 7-8 nm thick electron dense layer showing a transverse periodicity of about 7 nm. 

The major challenge to elucidating the structure of the desmosomal plaque is its density. The most effective method of analysis thus far has been immunogold electron microscopy [79, 82–84]. The most detailed study used quantitative analysis of immunogold labelling of the N- and C-termini of DP, PG, and PKP 1 and the C-termini of Dsg 3, Dsc 2a, and Dsc 2b to produce a molecular map of the desmosomal plaque [8]. This showed that the ODP is the region where the armadillo proteins probably interact with the N-terminus of DP and the cytoplasmic domains of the desmosomal cadherins while the C-terminus of DP lies in the IDP in accordance with its known function of binding the IFs. Mapping the localisation data from the study of North et al. in [8] onto the structure reported by Al-Amoudi et al. [80, 81] suggests that the density Al-Amoudi et al. reported in the plaque corresponds to this same region of substantial protein-protein interaction.

## 12. Protein Kinase C Modulates Desmosomal Adhesiveness

Since modulation of desmosomal adhesion involves no change in protein composition, an alternative mechanism might involve some type of inside-out signalling such as has been found to modulate the adhesiveness of, for example, integrins [85, 86]. That such is also the case for desmosomes was indicated by the observations that treatment of MDCK and HaCaT cells with protein kinase C (PKC) activators rapidly converted desmosomes from hyperadhesive to calcium dependent [37, 54]. Conversely, PKC inhibitors induced hyperadhesiveness in calcium-dependent desmosomes. Because (i) MDCK cells contain relatively few PKC isozymes, (ii) an inhibitor of conventional PKC isozymes was effective in calcium-dependence/hyperadhesion conversion, and (iii) PKC was localised to the membranes of cells with calcium-dependent desmosom es but diffuse cytoplasmic in cells with hyperadhesive desmosomes, it was concluded that PKC plays an important role in this conversion [37]. Is this also the case in vivo? 

In normal mouse epidermis where all desmosomes appear to be hyperadhesive, PKC is most strongly expressed in the basal layer, has a diffuse cytoplasmic distribution, and shows no colocalisation with desmosomes. (N. B. PKC expression may differ between layers of the epidermis in humans and mice [87].) However, in wound-edge epidermis where the desmosomes are predominantly calcium dependent, PKC is abundantly localised to desmosomal plaques [55]. This strongly suggests that PKC plays a role in regulating desmosomal adhesiveness in vivo but does not constitute definitive functional evidence. 

During epidermal development calcium-dependent desmosomes at E12 could be converted to hyperadhesiveness by treatment with the conventional PKC isozymes inhibitor Gö 6976 suggesting that in vivo, as in culture, PKC is involved in desmosomal regulation (Kimura et al., unpublished). However, PKC*α* −/− mice developed normal desmosomes that showed developmental regulation to hyperadhesion with the same timing as wild-type mice, suggesting either that additional regulatory mechanisms are involved or that other isozymes compensate for the absence of PKC. If the above observations on the relocalisation of PKC in epidermal wound healing have functional significance, inhibition/activation of this isozyme should have predictable effects on wound healing and this is currently being tested. 

We were surprised to find recently that treatment of MDCK cells with the general tyrosine phosphatase inhibitor sodium pervanadate caused a substantial conversion of calcium-dependent desmosomes of subconfluent MDCK cells to hyperadhesion [88]. This treatment caused increased tyrosine phosphorylation of plakoglobin and desmoglein 2, which nevertheless remained in complex in the soluble cell fraction. The observation also clearly suggested that protein tyrosine kinases in addition to PKC may be involved in regulating desmosomal adhesiveness. 

How could protein kinases regulate desmosomal adhesiveness? Given the clear localisation of PKC to the desmosomal plaque during conversion of desmosomes, the most likely mechanism would seem to involve phosphorylation of one or more of the desmosomal plaque components. This could then cause a configurational change within the plaque that could in turn lead to a disordering of the plaque and a consequent disordering of the extracellular domains of the desmosomal cadherins leading to calcium dependence. Dephosphorylation would then restore order and lead to hyperadhesion. Such a mechanism would require no change in the major components of desmosomes, in accordance with our results, though it may require the recruitment and/or loss of protein kinases and phosphatases. The difficulty is in determining which desmosomal protein(s) is the key phosphorylation target. 

We and others have shown by metabolic labelling and immunoprecipitation that all major desmosomal components in cultured cells are phosphorylated under normal conditions [89, 90]. The phosphorylation may be on serine/threonine or tyrosine in the case of plakoglobin and desmoglein [88, 91–94]. The difficulty with such experiments lies in knowing the origin of the desmosomal components concerned; only phosphorylation changes that occur within desmosomes themselves can account for the adhesive changes. Changes that occur in soluble cell fractions are not relevant, though they may be associated with desmosome assembly. 

**Figure 6:**
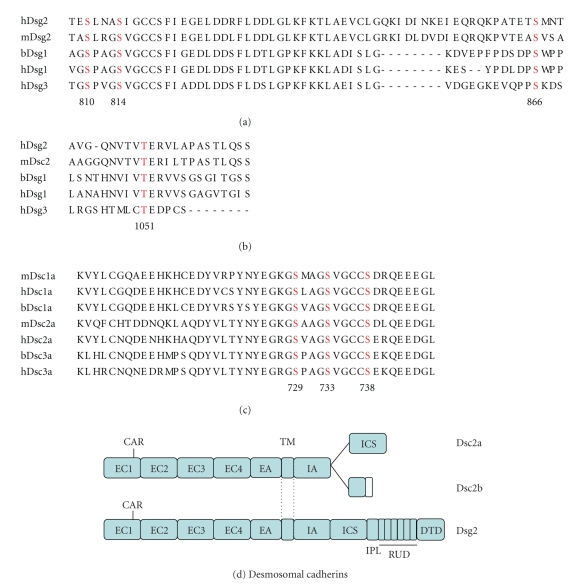
Mutations of phosphorylation sites in desmoglein and desmocollin produce slight increases in desmosomal hyperadhesion. (a) and (b) show conserved predicted phosphorylation site (in red) in the cytoplasmic domains of desmogleins and (c) shows similar sites in desmocollins. These were mutated to alanine and the mutant proteins expressed in MDCK cells. The effects on desmosomal adhesiveness are described in the text. (d) shows stick models of the desmosomal cadherins desmocollin 2 (Dsc2) and desmoglein 2 (Dsg2). Sites S810 and S814 are located towards the C-terminal end of the ICS (intracellular cadherin-specific) region of Dsg2, S866 towards the C-terminal end of the IPL (intracellular proline-rich) region, and S1051 in the sixth repeat of the specific repeat unit domain (RUD). Sites S729, S733, and S738 are towards the C-terminal end of the ICS region of Dsc2a but are absent from the shorter C-terminal region of Dsc2b because alternative splicing truncates this region. (d) is reproduced from the study by Garrod and Chidgey in [1].

An alternative approach is mutagenesis of known or potential phosphorylation sites. Quantitative analysis of the location of PKC in the plaques of epidermal wound-edge desmosomes suggested a biphasic distribution, one peak lying within a few nanometers of the inner leaflet of the plasma membrane and the other within the outer dense plaque [55]. Based on our previous molecular mapping of the desmosomal plaque, no desmosomal component could be eliminated on the basis of colocalisation with PKC [8]. Accordingly we undertook mutagenesis of conserved consensus phosphorylation sites in the cytoplasmic domains of desmocollin 2 and desmoglein 2, the desmosomal cadherins with the widest tissue distributions, reasoning that sites involved in such a key regulatory process would be likely to be conserved (A. Smith, S. Haddad, Z. Nie, and D. Garrod; unpublished) (Figure 6). Mutant proteins were expressed in MDCK cells and the cells assessed at an early stage of confluent culture for increased hyperadhesiveness. It was found that the combined mutations S729A and S738A in Dsc2 and S810A and T1051A in Dsg2 increased the percentage of sub-confluent/early confluent cells with hyperadhesive desmosomes to 22% compared with 6% for cells transfected with plasmids encoding wild-type proteins. RNAi depletion of endogenous Dsg2 increased this figure to about 37%. Furthermore, both mutations significantly slowed the rate of healing in a scratch wound assay. These rather modest effects probably indicate that regulation of desmosomal adhesiveness is a complex process involving multiple sites on several desmosomal proteins.

## 13. Downregulation of Desmosomes In Vivo: Internalisation versus Disassembly

A vital aspect of tissue homeostasis is the turnover of molecules and organelles. In the case of desmosomes in epithelial tissues such as the epidermis, the need for such turnover is clear. As keratinocytes migrate upwards through the epidermal layers, the protein composition of individual desmosomes changes, the more basally expressed desmosomal cadherin and plakophilin isoforms being gradually replaced by others that are upregulated in the upper layers [1, 10], while the other components, desmoplakin and plakoglobin, which do not have multiple isoforms, continue to be expressed. It is not known how this change is achieved; it could be by molecular exchange within existing desmosomes, as seems possible in cultured cells [47], or by downregulation of existing desmosomes and replacement with new ones of differing composition. Of these two alternatives, there is no evidence for the former in vivo, while there is evidence for the latter, not as far as I know from intact, normal epidermis, but from situations where desmosome downregulation would be expected to be increased, such as wound healing and disease. 

Downregulation of desmosomes in wound-edge epidermis appears to have been first reported by Croft and Tarin [95]. It seems intuitively obvious that for multilayered keratinocytes to form a single migrating layer they must reduce their intercellular adhesions. Reduce but not abolish, because maintenance of cell-cell adhesion appears essential for coordinated migration of epithelial cell sheets [96]. When examining wound-edge epidermis we were surprised that we never found half desmosomes [55]. (Apparently desmosome splitting occurs when calcium is chelated but does not appear to be the “normal” methods of downregulation.) Instead, whole, apparently intact desmosomes were frequently observed inside wound-edge cells. This suggests that engulfment of entire desmosomes may be the mechanism of downregulation, consistent with a mechanism for desmosomal engulfment previously proposed by Allen and Potten [97]. Membrane processes originating from one of a pair of cells were suggested to engulf the desmosome enclosing it in a cytoplasmic vesicle within the protagonist. What determines which member of the cell pair gets the desmosome is not known. Such a mechanism would be consistent with the finding that the actin cytoskeleton is involved in internalisation of half desmosomes in cultured cells [98]. The puzzle in our studies was that the majority of internalized desmosomes did not appear to be enclosed in vacuoles, and the reason for this is not clear. Internalised desmosomes, usually with at least a partial associated vacuole, have been reported from a variety of skin lesions (see [55] for references) but it is not clear whether this is the general mechanism for desmosome downregulation in vivo. 

Desmosome downregulation is frequently referred to as desmosome “disassembly”, largely on the basis of research on cultured cells. “Disassembly” implies the dissolution or disruption of an object into its component parts or in this case its component molecules. In order for this to be conclusively demonstrated it has to be shown that molecules appearing in the soluble fraction of cells were previously incorporated into desmosomes. The author submits that this is rarely, if ever, the case. Comparison between the non-ionic detergent-soluble and insoluble fractions is a start but is in itself inadequate because some desmosomal components enter the insoluble fraction shortly after synthesis and probably before incorporation into desmosomes [99]. Thus the difference between the soluble and insoluble fractions could represent prevention of assembly rather than promotion of disassembly. Furthermore, in the lack of any convincing evidence to the contrary, such studies have invariably been carried out on immature, calcium-dependent desmosomes, the in vivo relevance of which is doubtful. There is no evidence that the author is aware of that desmosome disassembly ever occurs in vivo. 

Even when calcium-dependent desmosomes are disrupted in culture by chelation of extracellular calcium, there is no evidence that they disassemble. Under these circumstances desmosomes split in the midline (Figure 4) and the resulting half desmosomes are internalised into cytoplasmic vacuoles [49, 50]. Such half desmosomes are gradually transported to the perinuclear region of the cell and continue to be so even if a new round of desmosome assembly is initiated by once again restoring extracellular calcium [47, 50]. This suggests that desmosomes are not disassembled and their components reutilised for the assembly of new desmosomes. Our unpublished studies based on immunofluorescent colocalisation of all major components of internalised desmosomes and their insolubility in internalising cells reaffirm that internalised desmosomal halves do not disassemble (McHarg, Hopkins, Lin, and Garrod; unpublished).

## 14. Some Implications for and Suggestions from Human Disease

In pemphigus vulgaris (PV), a potentially fatal blistering disease of skin and mucous membranes, autoantibodies to the desmosomal cadherins Dsg3 (and Dsg1 in some patients) cause loss of cell-cell adhesion or acantholysis [100, 101]. Suggested alternative but not necessarily mutually exclusive mechanisms for acantholysis include direct disruption of desmosomal adhesion due to steric hindrance by the autoantibodies and activation of outside-in signalling by autoantibody binding [16, 102]. 

A review of the literature on the ultrastructural analysis of PV appears to show that direct disruption of desmosomal adhesion is not the primary event [103–107]. Rather there is extensive loss of cell-cell adhesion in interdesmosomal regions and possible intracellular cleavage behind the desmosomal plaque that might indicate a weakening of the cytoskeleton, perhaps through a signalling mechanism involving plakoglobin [102, 103]. By contrast abundant split desmosomes with inserted keratin filaments were found in a mouse model of PV [108]. This model involves immunising desmoglein 3 null mice against desmoglein 3 and then transferring splenocytes from the immunised mice to Rag2 null mice, which then produce antidesmoglein 3 and develop symptoms of PV [109]. Ingenious though this model is, the above results on disruption of desmosomal adhesion should be regarded with caution because there is no guarantee that the antibodies produced function by the same mechanism as PV autoantibodies. 

A series of publications reporting work in which keratinocytes were treated with PV autoantibodies in culture showed that desmosomes are downregulated and that desmoglein 3 is depleted and internalised [110–115]. However, all of this work was presumably carried out with keratinocytes possessing calcium-dependent desmosomes, which, as we have shown, do not represent the in vivo situation. A recent study in which comparison was made between keratinocytes with calcium-dependent desmosomes and those with hyperadhesive desmosomes showed that hyperadhesion substantially inhibited PV autoantibody-induced acantholysis and internalisation of adhesion molecules including desmoglein 3 and E-cadherin [116]. Further work is needed to determine the extent to which hyperadhesive keratinocytes in culture provide a suitable model for PV but the results of Cirillo et al. are extremely encouraging. 

While the primary effect of PV autoantibodies does not appear to be direct disruption of existing desmosomal adhesion, they could inhibit de novo desmosome assembly, which must be a continuous process in stratified epithelia [117]. During the progress of disease this would be expected to result in gradual downregulation of desmosomes and loss of cell-cell adhesion. It would therefore be interesting to know whether the abovementioned results obtained with calcium-dependent keratinocytes in culture have any counterpart in vivo. It could also be the case that the primary loss of inter-desmosomal adhesion found in PV might resemble epidermal wounding and thus cause activation of PKC and consequent weakening of desmosomal adhesion as we have described [55]. In this case inhibition of PKC could provide a novel therapy for Pemphigus, as Cirillo et al. and others have suggested [116, 118]. 

Does human disease provide any suggestions as to how hyperadhesion might be regulated? A certain amount of speculation on this point appears possible. 

A single case of lethal acantholytic epidermolysis bullosa has been described [119]. This was caused by the occurrence of two different recessive mutations in the carboxy-terminal domain of desmoplakin resulting in the loss of IF attachment to desmosomes, a consequence that is entirely consistent with molecular cell biological studies on the role of desmoplakin in IF attachment [32, 120–123]. This resulted in massive epidermal acantholysis during birth, not because of loss of desmosomal adhesion, but because of cellular disruption behind desmosomal plaques [119]. Careful examination of the electron micrographs in the latter paper suggests that, though lacking IFs, the desmosomes were otherwise perfectly formed including a normal plaque structure and a clear midline in the intercellular space. If we are correct in our suggestion that the presence of a midline is consistent with hyperadhesion, this would indicate that hyperadhesion does not depend on desmosomal interaction with the IF cytoskeleton. 

Ectodermal dysplasia-skin fragility syndrome, the first human disease shown to result from a mutation in a desmosomal component, results from a mutation in the gene for plakophilin-1 [124]. Effectively a plakophilin-1 knockout, this condition results in skin blistering, complete absence of hair, and pachyonychia and is nonlethal probably because of compensation by other plakophilin isoforms. There are small epidermal desmosomes with absence of midline and inner dense plaque, and detachment of IFs. Keratinocytes isolated from diseased epidermis fail to develop desmosomal resistance to depletion of extracellular calcium and thus hyperadhesion [125]. This suggests that plakophilin-1 contributes to the development of desmosomal hyperadhesion. Similarly, mouse keratinocytes lacking plakoglobin do not develop hyperadhesion (McHarg, Müller, and Garrod; unpublished observations). 

The above observations together with our afore-mentioned mutagenesis of desmosomal cadherins suggest that the establishment and regulation of hyperadhesion may be a complex process involving more than one desmosomal component and more than one kinase. An experimental investigation of the molecular basis of hyperadhesion and its regulation is currently in progress.
